# Rasch analysis of the Meaning in Life Questionnaire among adults from South Africa, Australia, and New Zealand

**DOI:** 10.1186/s12955-016-0414-x

**Published:** 2016-01-20

**Authors:** Lusilda Schutte, Marié P. Wissing, Suria M. Ellis, Paul E. Jose, Dianne A. Vella-Brodrick

**Affiliations:** Africa Unit for Trans-disciplinary Health Research, North-West University, Potchefstroom, South Africa; Statistical Consultation Services, North-West University, Potchefstroom, South Africa; School of Psychology, Victoria University of Wellington, Wellington, New Zealand; Melbourne Graduate School of Education, University of Melbourne, Melbourne, Australia

**Keywords:** Rasch modelling, Meaning in Life Questionnaire, Eudaimonic well-being, Psychometric properties, Modern psychometrics, Item response theory, Positive psychology, Quality of life

## Abstract

**Background:**

Meaning in life is a key indicator of subjective well-being and quality of life. Further developments in understanding and enhancing the construct will depend inter alia on the sound measurement thereof. This study is at the forefront of applying modern psychometric techniques to the Meaning in Life Questionnaire, a scale widely used to assess meaning in life.

**Method:**

The Rasch rating scale model was applied to the Presence and Search subscales of the Meaning in Life Questionnaire using a sample of 601 adults from South Africa, Australia, and New Zealand.

**Results:**

The Presence subscale was insensitive at high levels of presence of meaning while the majority of the respondents fell in that range. Removal of item 9 (“My life has no clear purpose”) and collapsing the response categories indicative of low and medium levels of the latent construct significantly improved the subscale’s targeting and fit to the Rasch model, resulting in a subscale that exhibited differential item functioning on items 1 (“I understand my life’s meaning”), 4 (“My life has a clear sense of purpose”), and 5 (“I have a good sense of what makes my life meaningful”) for country, but none for gender, age group, or education level. The Search subscale yielded disordered category threshold calibrations, but after collapsing some of the response categories representing low and medium levels of the target construct, a subscale that demonstrated good fit to the Rasch model, good targeting, and no differential item functioning resulted.

**Conclusions:**

In terms of this particular scale, adaptation of the rating scale and removal of item 9 is recommended. Country-level parameter estimates may be needed for items that exhibited differential item functioning. The study also has significant implications for the theory, measurement, and practice of meaning in and quality of life in general. Reasons for and the far-reaching implications of the insensitivity of the Presence subscale for high levels of presence of meaning on, for example, the correlation between meaning in life and indicators of health are contemplated. Further investigation of the construct’s nature and measurement, especially at high levels, is indicated.

## Background

Quality of life involves an evaluative judgement of an individual’s physical, cognitive, emotional, and social functioning and can be based on subjective (self-report) and/or objective (independent sources of information) indicators [1, 2]. Although quality of life research traditionally focused on situations and factors that undermine or endanger quality of life, recent research has increasingly stressed the importance of incorporating positive constructs, such as subjective well-being, positive emotions, and character virtues and strengths in the conceptualisation and study of quality of life [2, 3]. One of the key constructs that is widely considered an integral part of a life well-lived and quality of life is meaning in life [4–7]. A myriad of studies have explored the relationship between meaning in life and mental well-being, as well as psychopathology [8]. Also, the association between meaning in life and health-related quality of life has been established in multiple studies [9].

In order to study meaning in life and its quality of life concomitants, the construct has to be conceptualised theoretically. Different models are used in the literature to conceptualise this complex phenomenon, for example those of Wong [10], Schnell [11], and Steger [12]. Steger’s model differentiates between presence of meaning, which involves “the extent to which people comprehend, make sense of, or see significance in their lives, accompanied by the degree to which they perceive themselves to have a purpose, mission, or overarching aim in life” [12], and search for meaning, which refers to “the strength, intensity, and activity of people’s desire and efforts to establish and/or augment their understanding of the meaning, significance, and purpose of their lives” [13].

Theoretically and empirically sound measurement instruments that assess meaning in life are crucial for the rigorous study of the construct, to understand its associations with psychological well-being and psychopathology, and to assess the impact of interventions targeting meaning in life. Various models of meaning have been operationalized in self-report questionnaires (see [14] for a systematic review of these measures). One such scale that is widely used and recognized for its outstanding psychometric properties [14] is the Meaning in Life Questionnaire (MLQ) [15], which operationalizes Steger’s [12] model of meaning in life. Steger et al. [15] showed that the scale, which consists of two subscales corresponding to the theory, namely Presence of Meaning (MLQ-P) and Search for Meaning (MLQ-S), demonstrated sufficient internal consistency and test-retest reliability, as well as structural, convergent, and discriminant validity in three American student samples.

Since the initial development study of the MLQ [15], which utilised only data from American student samples, good psychometric properties of the scale have been shown in a number of other contexts, cultures, and translations. For example, validity and reliability were shown for the English version of the scale among a web-based survey of adults [16], an American sample of people diagnosed with serious mental illnesses in an inpatient setting [17], and in a multi-cultural South African student setting [18]; for the Japanese translation of the scale among a Japanese student sample [19]; for the Spanish translation of the scale among a Spanish student sample [20]; and for the Turkish version of the scale among a combined college student and adult community sample [21].

Even though the MLQ is widely appraised to possess good psychometric properties [14] and the measure has been found to function well across age groups [13] and cross-culturally [13, 21, 22], the scale has, as far as we are aware of, never been evaluated from an item response theory (IRT) perspective. IRT provides a modern and reputedly superior alternative to classical test theory, as it discriminates more finely among different sources of error, especially regarding features of individual items that may influence their performance [23]. The family of IRT models share the assumption that the probability of a respondent endorsing any particular item is considered to be a function of the respondent’s level on the underlying latent variable that is measured and the characteristics of the item [24].

The Rasch model, specifying only one parameter to characterize each item (item difficulty), is the simplest IRT model and was developed by the Danish mathematician, Georg Rasch [25, 26]. Unlike in other IRT models and classical test theory techniques where the intent is to find a model that best fits the data, the Rasch model requires the data to fit the model in order to yield objective measurement [27]. The Rasch model postulates that useful measurement involves a unidimensional construct increasing or decreasing monotonically along an interval scale [28]. Rasch modelling provides a method to transform ordinal data (e.g. data from Likert-type items) into continuous, equal interval units (logits), which allows for the summation of the items’ raw scores, where the summed raw score is a sufficient statistic [29, 30]. Rasch analysis can be used in scale development, for example by reviewing the functioning of the response categories, the unidimensionality of the scale, and the targeting of the measure [31]. Moreover, Rasch modelling can be used to investigate differential item functioning (i.e., when different demographic groups responded differentially to an item despite equal levels of the latent construct), thus enhancing the assessment of item-level cross-cultural invariance of measurement scales [32].

### The present study

In the present study, the Meaning in Life Questionnaire [15] was examined against the assumptions of the Rasch model. This is the first known study where the scale is analysed using an item response theory (in particular, Rasch modelling) approach. By applying the Rasch model, we explored the unidimensionality of each subscale, the functionality of the response categories, and how well the sample was targeted by the scale. We also examined differential item functioning (DIF) of the scale for a range of demographic variables.

## Method

### Participants

The sample (*N* = 601) consisted of about equal sized groups of adults from South Africa, New Zealand, and Australia, who all completed the original English version of the MLQ as part of a battery of scales used in the international Eudaimonic and Hedonic Happiness Investigation (EHHI) project [33]. Participants were selected to be fluent in English, have at least secondary education, and be between 30 and 60 years of age. The aim was to factorially cross gender, age (three age groups of 30-39 years, 40-49 years, and 50-60 years), and education level (secondary and tertiary education). The socio-demographic profile of the sample is summarised in Table 1.

**Table 1:** Demographic profile of the sample

	South Africa	New Zealand	Australia	Total
Gender				
Male	101	107	79	287
Female	115	108	91	314
Age				
*M* (*SD*)	44.11 (8.53)	44.45 (8.85)	44.62 (8.84)	44.38 (8.72)
30-39	77	71	53	201
40-49	71	72	58	201
50-60	68	72	59	199
Education level				
Secondary	106	106	68	280
Tertiary	106	109	102	317
Missing	4	0	0	4
Total	216	215	170	601

### Measures

#### Socio-demographic questionnaire

Demographic information of each participant, including country of residence, gender, age group, and education level, was obtained.

#### Meaning in Life Questionnaire (MLQ) [15]

The MLQ comprises two subscales that was developed to be relatively independent: Presence of Meaning (MLQ-P) and Search for Meaning (MLQ-S) [15]. Responses to 10 statements are provided on a rating scale with response options 1 = *Absolutely Untrue*, 2 = *Mostly Untrue*, 3 = *Somewhat Untrue*, 4 = *Can’t Say True or False*, 5 = *Somewhat True*, 6 = *Mostly True*, and 7 = *Absolutely True.* In the original validation study among American students, the scale exhibited good internal consistency and test-retest reliability, as well as structural, convergent, and discriminant validity, with the Cronbach’s alpha values of the Presence subscale varying between 0.82 and 0.86 and for the Search subscale between 0.86 and 0.87 [15]. Good internal consistency reliability was found in South African student [18], New Zealand adult [34], and web-based Australian samples [35], with alpha-values of .85, .90, and .88, respectively, for the MLQ-P, and .94, .91, and .92, respectively, for the MLQ-S.

### Procedure and ethical considerations

A mixed-methods cross-sectional survey design was used, where participants responded to open-ended questions related to happiness, meaning in life, and goals, and completed a battery of quantitative measurement scales. For the current investigation, only responses to socio-demographic questions and the MLQ were used. In order to avoid the potential complications of missing values and imputation techniques in Rasch analyses, respondents who generated missing values on the MLQ were removed from the sample. This involved 15 participants from South Africa, whose removal was justified by the fact that the original South African sample was larger than the samples from Australia and New Zealand. The sample from New Zealand contained no missing responses, and for the Australian sample six respondents were removed. Ethical approval was obtained from the respective regulatory ethics committees in each country. Participants were recruited by research leaders within each country using poster and newspaper advertisements and the snowball-method. Participants were provided with information on the study prior to voluntary participation.

### Data analysis

Data were analysed using the Rasch rating scale model [25], which assumes that the distances between the thresholds of polytomous items (i.e., the probabilistic midpoints between adjacent response categories) are equal across all items. The Winsteps® 3.81 software [36] was used for all analyses, except for the graphical presentation of the person-item threshold distributions (Fig. 2), which was obtained from RUMM2030™ [37]. The MLQ-P and MLQ-S were evaluated separately, since the scale was designed to yield two relatively independent subscales [15]. Since no single aspect of Rasch analysis is definitive in identifying the optimal data-model relationship, multiple tests and graphical representations should be used to examine the characteristics of the items and persons [30]. The following interrelated facets of Rasch analysis should be considered simultaneously to inform decisions.

#### Person and item separation and reliability

Person separation and reliability indices indicate how well one can discern persons along the measured variable [28] and values larger than 2 and 0.8, respectively, imply that the items are sensitive enough to differentiate two levels of persons according to their level of intensity on the construct (high and low scorers) [38]. Item separation and reliability indices are indicative of the capacity of the instrument to define a unique hierarchy of items along the measured construct [28] and values larger than 3 and 0.9, respectively, suggest that the sample is large enough to confirm the item challenge order (on three levels of item challenge) [38].

#### Unidimensionality and local independence

According to the Rasch model, useful measurement is obtained when a unidimensional construct is measured by locally independent items [30]. In terms of unidimensionality, item infit or outfit mean square statistics smaller than 0.6 can be indicative of overfit, and values larger than 1.4 of underfit when the rating scale model is used [28]. The point-biserial correlation of an item indicates whether higher scores on the item correspond with higher levels of the underlying construct and positive values are expected [38]. In addition, lack of unidimensionality may exist when the eigenvalue of the first contrast in a Rasch principal components analysis of the residuals (PCA-R) (i.e., the first component after the Rasch component has been removed) is larger than 2.0, and when the variance explained by the Rasch component is small (e.g., < 40 %) [38]. Correlations between the residuals of item pairs of around 0.7 are indicative of high local dependence, while correlations around 0.4 are considered to be low [38].

#### Response category functioning

Rasch analysis enables the researcher to investigate how the respondents used the rating scale so that scale developers can decide on the optimal number and combination of rating scale categories [31, 39]. This task can be accomplished by examining how the data fit the Rasch model after response categories were collapsed. Bond and Fox [28] provided guidelines in this regard, including that the collapse should make intuitive sense and that the ideal is to create a uniform frequency distribution over the categories with each category containing at least 10 observations. Also, the average measures of the categories and the category threshold estimates should increase monotonically, with the category threshold estimates having steep gradients (at least 1.4 logits, but no more than 5.0 logits) to ensure that each category represents a distinct portion of the latent variable – this can also be investigated graphically by looking at the category probability curves. Lastly, the infit and outfit mean square statistics of each response category should be less than 2.0.

#### Targeting

Rasch analysis can be used to detect gaps in the continuum of the measured construct by identifying poor targeted items or persons, such as items for which there is an insufficient number of persons with an intensity level comparable to the item challenge^1^, or persons for which there is an insufficient number of items with a challenge level comparable to the person’s intensity [40]. This goal can be attained by examining the person-item threshold distributions generated by RUMM2030™, which offers a visual comparison of the distribution of the person intensity levels (top part of the graph) and the item challenge levels (bottom part of the graph) along the latent trait continuum, with the information provided by the items also mapped onto the person distribution.

#### Differential item functioning

Rasch analysis can assist in identifying differential item functioning (DIF), which occurs when different groups of people within the sample responded in a different way to an item despite equal levels of the construct that was measured. In this study, uniform DIF [31] was investigated for country, gender, age group, and education level. The degree of DIF was assessed by comparing *p*-values from the polytomous version of the Mantel-Haenszel statistic [41, 42] against a Bonferroni-corrected 5 % significance level, as well as the DIF Contrast, which is indicative of moderate to large DIF when it is larger than or equal to 0.64 [38].

## Results

### Results for the presence subscale

#### MLQ-P

**Fig. 1:**
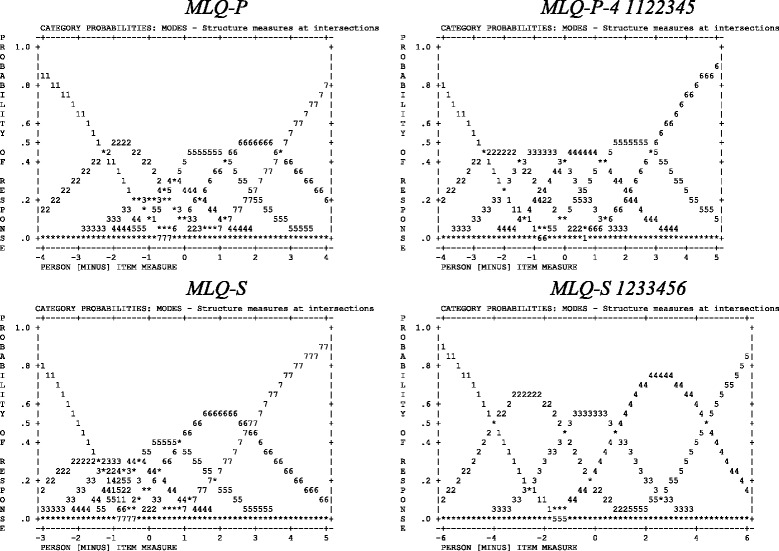
Category probability curves of the Meaning in Life Questionnaire (MLQ). MLQ-P = Original Presence of Meaning subscale of the MLQ; MLQ-P-4 1122345 = MLQ-P-4 with response categories 1 and 2 collapsed and categories 3 and 4 collapsed. MLQ-S = Original Search for Meaning subscale of the MLQ; MLQ-S 1233456 = MLQ-S with response categories 3 and 4 collapsed

**Fig. 2:**
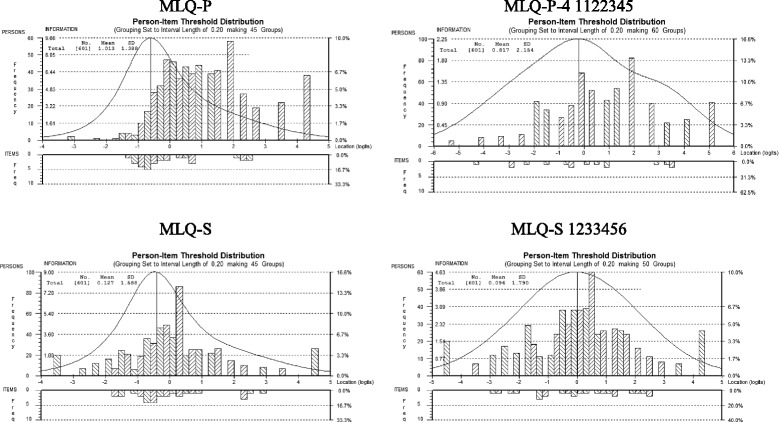
Person-item threshold distributions of the Meaning in Life Questionnaire (MLQ). MLQ-P = Original Presence of Meaning subscale of the MLQ; MLQ-P-4 1122345 = MLQ-P-4 with response categories 1 and 2 collapsed and categories 3 and 4 collapsed. MLQ-S = Original Search for Meaning subscale of the MLQ; MLQ-S 1233456 = MLQ-S with response categories 3 and 4 collapsed. Person-item threshold distributions were obtained from RUMM2030™

Although the MLQ-P yielded person and item separation and reliability indices that were in line with the guidelines and the results from the PCA-R suggested sufficient unidimensionality and local independence of the items (see Table 2), item 9 (“My life has no clear purpose”) showed misfit based on its infit and outfit mean square statistics (see Table 3). Also, response category 1 (*Absolutely untrue*) exhibited a low frequency and misfit based on its outfit mean square statistic (see Table 4). Although the average measures and threshold calibrations increased monotonically as the categories increased, the threshold calibrations were close to each other, indicating that categories 2 (*Mostly untrue*), 3 (*Somewhat untrue*), and 4 (*Can’t say true or false*) were the most likely to be endorsed on only a small portion of the latent construct (see Table 4 and Fig. 1). From the person-item threshold distribution (Fig. 2) it was clear that the person intensity was in general higher than the item challenge, indicating that the scale exhibited poor targeting for persons with high levels of the latent construct. The MLQ-P showed DIF for country on items 1 (“I understand my life’s meaning”), 4 (“My life has a clear sense of purpose”), and 9 (“My life has no clear purpose”), as depicted in Table 6. There was no significant DIF for gender, age group, or education level.

**Table 2:** Separation, reliability, fit, and dimensionality by subscale and analysis

Analysis	Separation		Reliability		Infit MNSQ		Outfit MNSQ		Dimensionality and local independence		
	Person	Item	Person	Item	M	SD	M	SD	Eigenvalue of 1^st^ contrast	% Variance explained	Max residual *r*
MLQ-P	2.00	3.84	.80	.94	1.01	0.57	1.01	0.59	1.7	59.3	.04
MLQ-P-4	2.42	5.81	.85	.97	0.99	0.16	0.96	0.14	1.6	69.7	None
MLQ-P-4 1122345	2.41	5.91	.85	.97	0.99	0.14	0.98	0.14	1.6	69.1	None
MLQ-P-4 1222345	2.43	5.71	.86	.97	0.99	0.14	0.96	0.14	1.4	69.0	None
MLQ-S	2.45	5.90	.86	.97	0.99	0.17	0.97	0.17	1.6	68.7	None
MLQ-S 1223345	2.50	5.59	.86	.97	0.99	0.17	0.99	0.17	1.6	63.5	None
MLQ-S 1233456	2.52	5.74	.86	.97	0.99	0.16	0.98	0.99	1.6	66.5	None
Ideal values	>2	>3	>0.8	>0.9	<1.4	Small	<1.4	Small	<2	>40 %	<0.2

Note. *MNSQ* mean square statistic, *Eigenvalue of 1* ^*st*^ *contrast* eigenvalue of the 1^st^ contrast in the Rasch principal components analysis of the residuals, *% Variance explained* % variance explained by the Rasch component in the Rasch principal components analysis of the residuals, *Max residual r* maximum positive residual correlation, *MLQ-P* original Presence of Meaning subscale of the Meaning in Life Questionnaire, *MLQ-P-4* MLQ-P with item 9 removed, *MLQ-P-4 1122345* MLQ-P-4 with response categories 1 and 2 collapsed and categories 3 and 4 collapsed, *MLQ-P-4 1222345* MLQ-P-4 with response categories 2, 3, and 4 collapsed, *MLQ-S* original Search for Meaning subscale of the Meaning in Life Questionnaire, *MLQ-S 1223345* MLQ-S with response categories 2 and 3 collapsed and categories 4 and 5 collapsed, *MLQ-S 1233456* MLQ-S with response categories 3 and 4 collapsed

**Table 3:** Meaning In Life Questionnaire: item measures, standard errors, and fit statistics

	δ	SE	Infit	Outfit	δ	SE	Infit	Outfit	δ	SE	Infit	Outfit
	MLQ-P				MLQ-P-4				MLQ-P-4 1122345			
1. I understand my life’s meaning.	0.09	0.04	0.92	0.90	0.10	0.06	1.23	1.17	0.09	0.07	1.21	1.20
4. My life has a clear sense of purpose.	0.17	0.04	0.60	0.62	0.23	0.06	0.79	0.80	0.31	0.07	0.83	0.82
5. I have a good sense of what makes my life meaningful.	-0.34	0.05	0.71	0.66	-0.61	0.06	1.04	0.97	-0.75	0.07	0.96	0.94
6. I have discovered a satisfying life purpose.	0.19	0.04	0.69	0.70	0.27	0.06	0.92	0.88	0.36	0.07	0.94	0.94
9. My life has no clear purpose.^a^	-0.12	0.05	2.13	2.18								
	MLQ-S				MLQ-S 1223345				MLQ-S 1233456			
2. I am looking for something that makes my life feel meaningful.	-0.30	0.04	1.14	1.11	-0.44	0.07	1.09	1.09	-0.36	0.05	1.11	1.10
3. I am always looking to find my life’s purpose.	-0.06	0.04	0.96	0.93	-0.12	0.07	0.96	0.96	-0.06	0.05	0.99	0.98
7. I am always searching for something that makes my life feel significant.	-0.20	0.04	1.05	1.03	-0.29	0.07	1.02	1.04	-0.24	0.05	1.02	1.04
8. I am seeking a purpose or mission for my life.	0.10	0.04	0.67	0.65	0.18	0.07	0.69	0.69	0.12	0.05	0.69	0.69
10. I am searching for meaning in my life.	0.46	0.04	1.12	1.12	0.67	0.07	1.19	1.17	0.54	0.05	1.13	1.11
Ideal values			>0.6 <1.4	>0.6 <1.4			>0.6 <1.4	>0.6 <1.4			>0.6 <1.4	>0.6 <1.4

Note. *MLQ* Meaning in Life Questionnaire, *MLQ-P* original Presence of Meaning subscale of the MLQ, *MLQ-P-4* MLQ-P with item 9 removed, *MLQ-P-4 1122345* MLQ-P-4 with response categories 1 and 2 collapsed and categories 3 and 4 collapsed, *MLQ-P-4 1222345* MLQ-P-4 with response categories 2, 3, and 4 collapsed, *MLQ-S* original Search for Meaning subscale of the MLQ, *MLQ-S 1223345* MLQ-S with response categories 2 and 3 collapsed and categories 4 and 5 collapsed, *MLQ-S 1233456* MLQ-S with response categories 3 and 4 collapsed. *δ* average item challenge, *SE* standard error of the item challenge, *Infit* infit mean square statistic, *Oufit* outfit mean square statistic

^a^The original item 9 was reversed in these analyses

**Table 4:** Meaning in Life Questionnaire – presence subscale: comparisons of the rating scale categories

	Observed count	Average measure	Infit	Outfit	Threshold calibration
MLQ-P					
1 – Absolutely untrue	79	-1.02	1.58	2.44	None
2 – Mostly untrue	111	-0.65	1.26	1.54	-1.47
3 – Somewhat untrue	208	-0.46	0.82	0.81	-1.19
4 – Can’t say true or false	352	-0.07	0.67	0.66	-0.67
5 – Somewhat true	700	0.64	0.67	0.60	-0.31
6 – Mostly true	919	1.71	0.87	0.89	0.83
7 – Absolutely true	636	2.55	1.39	1.19	2.82
MLQ-P-4					
1 – Absolutely untrue	56	-2.23	1.29	1.48	None
2 – Mostly untrue	73	-1.72	0.87	0.83	-2.59
3 – Somewhat untrue	152	-1.09	0.95	1.01	-2.09
4 – Can’t say true or false	288	-0.34	0.87	0.93	-1.32
5 – Somewhat true	633	0.83	0.74	0.74	-0.55
6 – Mostly true	810	2.85	0.96	0.91	1.49
7 – Absolutely true	392	4.56	1.75	1.21	5.05
MLQ-P-4 1122345					
1 (Original categories 1 and 2 collapsed)	129	-3.40	1.16	1.13	None
2 (Original categories 3 and 4 collapsed)	440	-1.82	0.94	1.00	-4.00
3 (Original category 5)	633	0.02	0.75	0.73	-1.22
4 (Original category 6)	810	2.20	0.92	0.94	0.78
5 (Original category 7)	392	3.98	1.44	1.27	4.43
Ideal values			<2	<2	

Note. *MLQ-P* original Presence of Meaning subscale of the Meaning in Life Questionnaire, *MLQ-P-4* MLQ-P with item 9 removed, *MLQ-P-4 1122345* MLQ-P-4 with response categories 1 and 2 collapsed and categories 3 and 4 collapsed, *MLQ-P-4 1222345* MLQ-P-4 with response categories 2, 3, and 4 collapsed, *Infit* infit mean square statistic, *Oufit* outfit mean square statistic

In an attempt to remedy the problems highlighted for MLQ-P, all possible combinations of response category collapses were explored, but none of the collapses resolved the problems with item 9. Therefore the next step was to remove item 9, resulting in a 4-item scale (hereafter labelled MLQ-P-4).

#### Results for the MLQ-P-4

The person and item separation and reliability indices improved significantly after item 9 was dropped from the scale (see Table 2). The PCA-R yielded results that confirmed satisfactory unidimensionality and local independence (Table 2) and all point-biserial correlations (values ranged between .79 and .85) and item infit and outfit mean square statistics (Table 3) pointed towards good fit. Although none of the response categories showed misfit based on their infit and outfit mean square statistics, the category probability curve (not shown) and threshold calibrations (see Table 4) still revealed that response categories 2 (*Mostly untrue*), 3 (*Somewhat untrue*), and 4 (*Can’t say true or false*) were the most likely to be endorsed over only a small portion of the latent variable, suggesting redundant response categories. Category 1 (*Absolutely untrue*) also still generated a low frequency. The person-item threshold distribution (not displayed) suggested even worse targeting for persons with high levels of the latent construct when compared to the full MLQ-P. The MLQ-P-4 showed DIF for country on items 1 (“I understand my life’s meaning”), 4 (“My life has a clear sense of purpose”), and 5 (“I have a good sense of what makes my life meaningful”) as depicted in Table 6. No significant DIF was found for gender, age group, or education level. In order to address the redundancy of the response categories, the next step was to explore all possible combinations of category collapses.

#### Results for the MLQ-P-4, response categories collapsed

Based on Rasch model diagnostics, two combinations of category collapses produced superior performance: One where category 1 (*Absolutely untrue*) was collapsed with category 2 (*Mostly untrue*), and category 3 (*Somewhat untrue*) with category 4 (*Can’t say true or false)* – hereafter labelled MLQ-P-4 1122345; and one where categories 2, 3, and 4 were collapsed – hereafter labelled MLQ-P-4 1222345. For both, the separation and reliability indices and the results from the PCA-R were in line with the results before collapsing categories (see Table 2). Due to space limitations only the results of the MLQ-P-4 1122345 are displayed in Tables 3, 4, and 5, and Figs. 1 and 2. Results for the MLQ-P-4 1222345 were similar, unless indicated in the text. The item infit and outfit mean square statistics (Table 3) and point-biserial correlations (values ranged between .83 and .88 for MLQ-P-4 1122345) indicated that all items fitted the Rasch model well and the response categories showed good fit, with threshold calibrations increasing monotonically and being sufficiently distanced from each other (see Table 4 and Fig. 1). For the MLQ-P-4 1222345, the frequency of category 1 (*Absolutely untrue*) was low, while the MLQ-P-4 1122345 yielded a larger frequency for category 1. Collapsing the categories improved the targeting of the scale considerably (see Fig. 2). Both the MLQ-P-4 1122345 and the MLQ-P-4 1222345 showed DIF for country on items 1 (“I understand my life’s meaning”) and 5 (“I have a good sense of what makes my life meaningful”) as shown in Table 6. No significant DIF was found for gender, age group, or education level.

**Table 5:** Meaning in Life Questionnaire – search subscale: comparisons of the rating scale categories

	Observed count	Average measure	Infit	Outfit	Threshold calibration
MLQ-S					
1 – Absolutely untrue	335	-1.99	1.53	1.52	None
2 – Mostly untrue	406	-1.45	0.72	0.77	-2.23
3 – Somewhat true	254	-0.55	0.83	0.84	-0.46
4 – Can’t say true or false	408	-0.07	0.75	0.67	-0.81
5 – Somewhat true	731	0.51	0.81	0.84	-0.38
6 – Mostly true	530	1.38	0.98	0.99	1.17
7 – Absolutely true	341	2.14	1.70	1.30	2.70
MLQ-S 1223345					
1 (Original category 1)	335	-3.11	1.43	1.39	None
2 (Original categories 2 and 3 collapsed)	660	-1.87	0.79	0.77	-3.55
3 (Original categories 4 and 5 collapsed)	1139	0.21	0.74	0.75	-1.27
4 (Original category 6)	530	1.72	0.91	0.91	1.64
5 (Original category 7)	341	2.68	1.37	1.43	3.18
MLQ-S 1233456					
1 (Original category 1)	335	-2.47	1.52	1.46	None
2 (Original category 2)	406	-1.80	0.69	0.71	-2.65
3 (Original categories 3 and 4 collapsed)	662	-0.39	0.78	0.76	-1.45
4 (Original category 5)	731	0.53	0.76	0.79	-0.06
5 (Original category 6)	530	1.52	0.96	0.99	1.25
6 (Original category 7)	341	2.35	1.55	1.43	2.90
Ideal values			<2	<2	

Note. *MLQ-S* original Search for Meaning subscale of the Meaning in Life Questionnaire, *MLQ-S 1223345* MLQ-S with response categories 2 and 3 collapsed and categories 4 and 5 collapsed, *MLQ-S 1233456* MLQ-S with response categories 3 and 4 collapsed. *Infit* infit mean square statistic, *Oufit* outfit mean square statistic

**Table 6:** Meaning in Life Questionnaire – presence subscale: differential item functioning for country

	MLQ-P (Bonferroni α = 0.003)					MLQ-P-4 (Bonferroni α = 0.004)					MLQ-P-4 1122345 (Bonferroni α = 0.004)				
Item	DIF Measure (DIF S.E.)			Contrast	MH	DIF Measure (DIF S.E.)			Contrast	MH	DIF Measure (DIF S.E.)			Contrast	MH
	AU	SA	NZ			AU	SA	NZ			AU	SA	NZ		
1	0.38 (0.08)	-0.28 (0.08)	0.16 (0.07)	AU > SA	AU > SA NZ > SA	0.36 (0.10)	-0.28 (0.11)	0.19 (0.09)	AU > SA	AU > SA	0.45 (0.13)	-0.41 (0.13)	0.25 (0.12)	AU > SA NZ > SA	AU > SA NZ > SA
4	0.32 (0.08)	-0.12 (0.08)	0.30 (0.07)		AU > SA NZ > SA	0.25 (0.10)	0.00 (0.10)	0.41 (0.09)		NZ > SA					
5						-0.76 (0.12)	-0.15 (0.11)	-0.89 (0.11)	SA > NZ	SA > AU SA > NZ	-0.97 (0.14)	-0.24 (0.12)	-1.07 (0.12)	SA > AU SA > NZ	SA > AU SA > NZ
9^a^	-0.79 (0.10)	0.41 (0.07)	-0.19 (0.08)	SA > AU	SA > AU NZ > AU SA > NZ										

Note. *MLQ-P* original Presence of Meaning subscale of the Meaning in Life Questionnaire, *MLQ-P-4* MLQ-P with item 9 removed, *MLQ-P-4 1122345* MLQ-P-4 with response categories 1 and 2 collapsed and categories 3 and 4 collapsed, *Bonferroni α* Bonferroni-corrected significance level, *DIF measure* item challenge for the particular country, *DIF S.E.* standard error of item challenge for the particular country, *Contrast* if the DIF contrast (i.e., the difference between the two countries’ DIF measures) was larger than or equal to 0.64, the countries are specified in this column, *MH* if the *p*-value of the Mantel-Haenszel test was smaller than Bonferroni α, the countries are specified in this column, *AU* Australian sample, *SA* South African sample, *NZ* sample from New Zealand. In columns DIF Contrast and MH, *x > y* implies that respondents from country *x* found it significantly harder to endorse the item than respondents from country *y* given equal levels of presence of meaning

^a^The original item 9 was reversed in these analyses

### Results for the search subscale

#### MLQ-S

The separation and reliability indices for the MLQ-S were in line with the guidelines, and the results from the PCA-R pointed to sufficient unidimensionality and local independence (see Table 2). Considering the item infit and outfit mean square statistics (Table 3) and the point-biserial correlations (values ranged between .80 and .85), all items fitted the Rasch model well. Although the infit and outfit mean square statistics of the response categories adhered to the guidelines, the threshold calibrations of categories 2 (*Mostly untrue*), 3 (*Somewhat untrue*), and 4 (*Can’t say true or false*) were disordered, pointing towards problematic use of the rating scale (see Table 5), which is also evident in the category probability curve (Fig. 1). The person-item threshold distribution (Fig. 2) portrayed that the average item challenge was slightly lower than the average person intensity, but from the information curve it is clear that there was substantial information available for the majority of respondents. There was no significant DIF for country, gender, age group, or education level. In an attempt to remedy the disordered threshold calibrations, all possible combinations of response category collapses were explored.

#### Results for the MLQ-S, response categories collapsed

Based on Rasch model diagnostics, two combinations of category collapses stood out as superior: One where category 2 (*Mostly untrue*) was collapsed with category 3 (*Somewhat untrue*), as well as category 4 (*Can’t say true or false*) with category 5 (*Somewhat true*) – hereafter labelled MLQ-S 1223345; and one where categories 3 (*Somewhat untrue*) and 4 (*Can’t say true or false*) were collapsed – hereafter labelled MLQ-S 1233456. Although the item separation dropped slightly after collapsing the categories, the person separation increased and the person and item reliability indices remained unchanged (see Table 2). Results of the PCA-R suggested sufficient unidimensionality and local independence (see Table 2). Based on the item infit and outfit mean square statistics (Table 3) and the point-biserial correlations (values ranged between .82 and .89 for MLQ-S 1223345 and between .82 and .88 for MLQ-S 1233456), all items manifested adequate fit. The problem of disordered category thresholds has been resolved, the distances between the threshold calibrations have improved, and the infit and outfit mean square statistics of the response categories pointed towards satisfactory fit (see Table 5 and Fig. 1). The person-item threshold distribution (Fig. 2) suggested improved targeting for MLQ-S 1233456, but for MLQ-S 1223345 (not shown) the average item challenge level was found to be more than the average person intensity level, which suggests less optimal targeting. There was no significant DIF for country, gender, age group, or education level.

## Discussion

Rasch analyses were conducted to investigate the psychometric properties of the Presence of and Search for Meaning subscales of the Meaning in Life Questionnaire, a scale which measures meaning in life – a key indicator of quality of life, from a Rasch modelling point of view. Data from three different countries were used. For MLQ-P, removal of item 9 (“My life has no clear purpose”) and collapsing response categories indicative of low and medium levels of the latent construct significantly improved the fit to the Rasch model and the targeting of the scale, resulting in a scale that exhibited DIF on items 1 (“I understand my life’s meaning”), 4 (“My life has a clear sense of purpose”), and 5 (“I have a good sense of what makes my life meaningful”) for country, but no DIF for gender, age group, or education level. The MLQ-S yielded disordered category threshold calibrations, but after collapsing some of the response categories representing low and medium levels of the target construct, a scale that demonstrated good fit to the Rasch model, good targeting, and no DIF resulted. Several specific aspects of the results will now be discussed.

### Reversed item

The first significant finding that warrants discussion is the poor performance of item 9 (“My life has no clear purpose”), the only reversed item in the MLQ-P scale. In a review on misresponse to reversed and negated items, Weijters and Baumgartner [43] advocated for the inclusion of reversed items in measurement scales as it can provide many benefits (e.g., control acquiescence, disrupt careless responding, and promote a broader coverage of the content domain), but stressed that it should be done with caution. A reversed item that is merely the negation of an item in the main direction (in point of fact, item 9 is basically the negation of item 4, “My life has a clear sense of purpose”), does not hold the benefit of broadening the content domain tapped by the instrument, and has the disadvantages inherent in negated items (e.g., accurately assessing level of agreement with statements that contain negation requires considerable cognitive strain) and reversed items (e.g., cross-cultural differences in response styles such as acquiescence). We therefore follow the guidance provided by Weijters and Baumgartner [43], who advised against the use of negated reversals, and consequently we recommend the removal of item 9, which will result in a 4-item Presence of Meaning subscale.

Steger et al. [15] stated that the reversed item was retained in the hope of discouraging automatic response sets. It is our view that this concern is to a large extent already handled by the mixed administration of the Presence and Search subscales. If item 9 is removed, however, the remaining items 4 to 6 will tap presence of meaning and the last three items will tap search for meaning. To guard against careless responding and response sets, we recommend shuffling the last six items (item 9 excluded) so that the respondent does not respond to three items from the same subscale in sequence.

### Number of response categories

For both subscales, the response categories indicative of low and medium levels of the latent construct appeared to be redundant and for the search subscale, the category thresholds were disordered. These findings suggest that the respondents were unable to distinguish reliably among the categories, and consequently fewer categories should yield more consistent, reliable scores. Weijters, Cabooter, and Schillewaert [44] suggested that seven response categories may be acceptable for populations who are expected to have high cognitive abilities, verbal skills, or questionnaire experience, such as college students, but that a 5-point scale may be more appropriate for the general population. For future use, we recommend either a 6-point rating scale where the midpoint category 4 = *Can’t say true or false* is dropped, or a 5-point scale with categories 1 = *Absolutely untrue*, 2 = *Untrue*, 3 = *Unsure*, 4 = *True*, 5 = *Absolutely true* (the issue of whether to include a midpoint category is much debated in the literature [44, 45]).

### Targeting

In the present study, the average level of meaning in life captured by the items was substantially lower than the average level of presence of meaning manifested by persons who completed the scale, suggesting poor targeting. In fact, the scale provided little information for respondents with high levels of presence of meaning while at the same time most of the respondents fell within that range. This could have significant practical implications. Correlations in correlational studies will be largely influenced by the minority of people exhibiting lower levels of presence of meaning as reflected by lower scores on the MLQ-P, while nuances of presence of meaning at the higher end of the continuum will not be captured well. This can, for example, influence outcomes of studies where the associations between meaning in life and indicators of health and quality of life are studied significantly. In addition, in experimental studies or studies where intervention programs are evaluated, the MLQ-P would probably not detect changes in meaning in life of people on the higher end of the continuum, which involves the majority of people, as the scale is not sensitive to changes at the higher end of the continuum.

Different explanations can be given for the findings regarding the targeting of the MLQ-P. One apparently obvious explanation is that there are not enough items or response options to capture high levels of the presence of meaning continuum and such items or response options should be added. However, given that the questionnaire already allows respondents to rate statements like “I understand my life’s meaning” to be “*absolutely true*”, it is not clear what kind of items or response options can be added to capture even higher levels of presence of meaning in life.

Another possible explanation pertains to the nature of presence of meaning as a construct and its distribution in the general population. The fact that the majority of the respondents endorsed high levels of presence of meaning according to their scores on the MLQ-P could simply tell us that most people indeed experience their lives as basically meaningful: Most respondents’ level of presence of meaning were higher than the levels where the scale had optimal information, merely because there is not much variability at the upper end of the underlying construct continuum. Such an explanation speaks to the findings of Heintzelman and King [46], who conducted a review of research on meaning in life from epidemiological data and studies using the MLQ-P [15] and the Purpose in Life Test [47]. They found that diverse samples rated themselves significantly above the midpoint on self-report measures of meaning in life and concluded that most people experience their lives as “pretty meaningful”. This line of thought can be linked to psychopathology literature where “quasi-traits” are distinguished. Reise and Waller [48] defines a quasi-trait as “a unipolar construct in which one end of the scale represents severity and the other pole represents its absence (depression versus not depressed)” which “is in contrast to a bipolar construct, where both ends of the scale represent meaningful variation (depression versus happiness)”. In psychopathology research, the existence of quasi-traits with their associated peaked information curves (with the peaks in the range representing severe levels of the trait) has been found in many item response theory applications and often led researchers to conclude that items needed to be added or adapted to provide information at low (less severe) levels of the trait continuum [48]. According to Reise and Waller [48] this reasoning is problematic when working with quasi-traits: If the underlying latent construct is a quasi-trait, such attempts may be futile – it will be difficult (if not impossible) to formulate items that yield information across the continuum of the trait. Similarly we can ask whether it would be possible to develop items designed to capture even higher levels of presence of meaning, or whether we should conclude that the variation of presence of meaning is limited at the higher end of the continuum, although the majority of people attain such high levels.

If we settle with the conclusion that the majority of the population attained maximum levels of presence of meaning, we will inevitably have to re-evaluate the usefulness of, for example, interventions that aim to enhance meaning in life in the general population (most of whom have attained high levels of meaning in life). The question would be what the (large) portion of people with high levels of meaning would gain from interventions that intend to enhance meaning. Accepting that the majority of the population have already attained levels of presence of meaning that do not allow for much improvement may pose further questions. For example, could it be possible that icons of eudaimonic living, such as Mahatma Ghandi, Mother Theresa, or Nelson Mandela, who sacrificed their lives for a greater cause, have experienced levels of meaning in life similar to the majority of people? Or should we rather conclude that the nuances of presence of meaning at higher levels are just not captured by the current conceptualisation and operationalization of the construct?

Another way to explain the poor targeting of the MLQ-P may be that the subscale applies a rather narrow understanding of meaning in life, with all items paraphrasing the notion of having found a sense of meaning or purpose in life. By repeating the same content using slightly different syntax, the scale actually operates in a similar way to a one or two-item measure, which could contribute to the inability of the scale to differentiate well at the higher end of the continuum. Alternative measures that capture a broader sense of meaning in life, such as the Sources of Meaning and Meaning in Life Questionnaire (SoMe) that operationalises meaningfulness through coherence, significance, direction, and belonging [11], may display better sensitivity.

In addition, one can argue that participants’ presence of meaning in life was not really as high as they indicated it to be – social desirability may have augmented their scores artificially. However, presence of meaning in life has been found to be unrelated to scores on measures of social desirability in several studies [15, 49] and, as argued by Heintzelman and King [46], high presence of meaning scores have been found consistently among diverse samples, including anonymous samples where social desirability may not have been a big concern. The high scores could have also been due to a generalisation effect – when asked to respond to items that concern global meaning in life, people may not be sure what meaning actually refers to. They may have a broad understanding of meaning and therefore think that they generally experience meaning. However, if the constituents of meaning are spelled out, they might realise that they don’t have as much meaning as they initially thought.

One may also reason that the lack of sensitivity to varying nuances of meaning in life at the higher end of the continuum relates to the fact that the scale relies on self-report and alternative avenues to capture meaning in life should be explored. This approach may be problematic because meaning in life is, at its heart, a subjective experience. Several studies have argued that self-report is the best way to capture meaning in life [46, 50, 51]. However, obtaining self-report using less structured approaches may add value, for example by using experience sampling methods [52] or qualitative methods.

### Differential Item Functioning (DIF)

The data in this study were gathered in three different countries and two gender groups, three age groups, and two levels of education were distinguished. Of all these demographic variables, significant DIF was only detected for items from the Presence subscale based on the country variable. The absence of DIF is the desirable outcome should data from the different demographic groups be combined or compared [53].

The significant country DIF for items from the Presence of Meaning subscale warrants further attention. Before removal of item 9 (“My life has no clear purpose”), the item exhibited DIF for country: Given equal levels of the latent trait, respondents from South Africa tended to respond more strongly towards the extreme *True* response categories than respondents from New Zealand and Australia, and, similarly participants from New Zealand were more inclined to extreme responses in the *True* direction than participants from Australia. After removal of item 9 and before collapsing the response categories, item 1 (“I understand my life’s meaning”) manifested DIF, where Australians found it harder to endorse the item than South Africans given equal levels of the latent construct. After collapsing categories, this finding was extended – respondents from both New Zealand and Australia found it significantly harder to endorse item 1 than respondents from South Africa given equal levels of the construct. Also, before collapsing categories, participants from New Zealand found it harder to endorse item 4 (“My life has a clear sense of purpose”) than participants from South Africa given equal levels of the latent trait. Last, given equal levels of the latent construct, participants from South Africa found it harder to endorse item 5 (“I have a good sense of what makes my life meaningful”) than respondents from Australia and New Zealand, both before and after collapsing categories. Country-specific parameter estimates may be needed for these items of the Presence subscale, that is, the dataset can be split by country and these items should be calibrated separately for each country [54].

The two items that respondents from Australia and New Zealand found harder to endorse than South Africans given equal levels of the latent construct (i.e., items 1 and 4) refer to comprehending one’s life meaning and having a clear sense of purpose – both can be seen as a global state of grasping one’s life meaning, without referring to the elements that brings meaning to one’s life. South Africa is a developing country and together with the many challenges the country faces come multiple opportunities for individuals to contribute and to have a sense of purpose. This may especially be the case for educated individuals who may feel that they have skills and knowledge that can really make a difference in a country with many challenges (based on the selection criteria of this study all participants had at least secondary education). Australia and New Zealand, on the other hand, are first world countries with a lot more stability and certainty. People from such countries may feel that things “go right” regardless of their contribution which may possibly lead to having a less clear sense of purpose and meaning comprehension. Another possible explanation may be connected with the fact that the specific South African group in this study exhibited a higher frequency of religious practice (mostly Christianity) than the participants from Australia and New Zealand. Religiosity may be associated with a clear sense of purpose and meaning comprehension.

The item that South Africans found harder to endorse than respondents from Australia and New Zealand given equal levels of the latent trait (item 5) refers to an awareness of the constituents of a meaningful life – the elements that make one’s life meaningful. One possibility is to infer that people (in this case, South Africans) who find it easier to agree with items referring to a global comprehension of one’s life’s meaning (items 1 and 4), may not have such a pressing need to know what the elements are that make their lives feel meaningful – one may argue that they take it for granted or that they spend less time attending to the specific details of why they find their lives meaningful. In contrast, people who find it more challenging to agree with items related to comprehending one’s life meaning and having a clear sense of purpose (in this case respondents from Australia and New Zealand), may be more attentive to the things that add life meaning.

For both items 4 and 9, South Africans tended to answer more strongly in the *True* direction when compared to respondents from Australia and New Zealand given equal levels of the latent construct. In other words, South Africans were more inclined to find both the non-reversed, non-negated statement “My life has a clear sense of purpose” (item 4) and the reversed, negated statement “My life has no clear purpose” (item 9) true. This points to a discrepancy which poses questions about the possible influence of response styles involved in responding to the reversed item that could have caused DIF. This finding provides additional support for the deletion of item 9.

Since all aspects of Rasch analysis are interconnected [30], the existence of cross-country DIF on the Presence subscale could have influenced the rest of the findings. Future research should explore whether the findings of this study replicate in more culturally homogeneous samples where DIF is not present.

### Limitations and future directions

While the study makes important contributions to the body of knowledge about meaning in life and the measurement thereof across three countries, it also possessed several limitations. This study made use of the Rasch model, which is considered to be a one-parameter IRT model that includes only item difficulty as a parameter. Although the Rasch model has very attractive mathematical properties, analysing MLQ data using more complex IRT models will also be of value.

In this study, recommendations regarding the removal of item 9 (“My life has no clear purpose”) and category collapses were made a posteriori based on removing the item from and collapsing categories of data attained using the original full scale. These recommendations should be tested in new datasets gathered with a revised scale.

The fact that the sample in this study comes from three different countries can be seen as a strength in the sense that diversity is reflected in the study of an already well-established scale. In addition, it allowed us to investigate DIF across the three countries. The fact that evidence was found for DIF across the countries, however, points towards the possibility that the scale may function differentially across the different country groups which could have had an influence on the rest of the results. This suggests the need for repetition of the study in more culturally homogeneous groups to investigate whether the findings replicate when cross-country influences are not present.

Another important avenue for future research is the revisiting of presence of meaning in life as a construct, in particular with regards to the higher end of the construct continuum. The content domain of presence of meaning should be explored qualitatively in order to deepen our understanding of the construct, especially at high levels. For example, by investigating lay people’s conceptualisations of meaning in life, we may identify sub-facets of meaning in life which may provide greater variance at the upper end of the continuum.

## Conclusions

The rigorous measurement of meaning in life is essential for the study of this key aspect of well-being and quality of life. The present study was the first to apply item response theory, in particular Rasch modelling, to investigate the psychometric properties and item-level equivalence of the MLQ across different demographic variables. The study offered valuable insights into the functioning of the MLQ in groups from South Africa, Australia, and New Zealand and the construct of meaning in life and the measurement thereof in general. In particular, the MLQ displayed good psychometric potential from a Rasch modelling perspective. However, several directions for revision were highlighted. First, the study pointed out that seven response categories may be too many when measuring meaning in life in the general population, and suggested that five or six response categories may be more appropriate. In addition, the study confirmed the potential problems involved in reversed, negated items, and suggested that this type of item should rather be avoided – removing the reversed phrased item 9 (“My life has no clear purpose”) was indicated. Although no DIF was found for the Search subscale, the Presence subscale displayed significant DIF for the country variable on four of its five items. Hypotheses were articulated to explore possible sources of the DIF, and it was suggested that country-level parameter estimates may be needed for these items. The existence of DIF pointed to the necessity of repeating this study in more mono-cultural settings to investigate whether the findings replicate. Furthermore, it was shown that people with high scores on presence of meaning were not targeted well by this highly commended meaning in life scale, while at the same time most of the respondents fell in that range. Reasons for and the extensive implications of this finding were contemplated and the vital importance of further exploration of the nature of the construct of presence of meaning and the measurement thereof, particularly at high levels, was indicated.

### Endnotes

^1^In the ability testing environment, where Rasch modelling originated, the term *item difficulty* is often used to refer to the level of the latent construct captured by the item, while *person ability* describes the level of the latent trait held by the respondent. For the purpose of the current study where meaning in life is studied, we deemed the terms *item challenge* and *person intensity* more suitable and used them in the manuscript*.*
